# *NFκB1* and *NFκBIA* Polymorphisms Are Associated with Increased Risk for Sporadic Colorectal Cancer in a Southern Chinese Population

**DOI:** 10.1371/journal.pone.0021726

**Published:** 2011-06-30

**Authors:** Shunxin Song, Dianke Chen, Jiachun Lu, Jiawei Liao, Yanxin Luo, Zuli Yang, Xinhui Fu, Xinjuan Fan, Yisheng Wei, Lei Yang, Lei Wang, Jianping Wang

**Affiliations:** 1 Gastrointestinal Institute of Sun Yat-Sen University, The Sixth Affiliated Hospital of Sun Yat-Sen University, Guangzhou, People's Republic of China; 2 Department of Colorectal Surgery, The Sixth Affiliated Hospital of Sun Yat-Sen University, Guangzhou, People's Republic of China; 3 Department of Gastrointestinal Surgery, The Second Affiliated Hospital of Guangzhou Medical College, Guangzhou, People's Republic of China; 4 The Institute for Chemical Carcinogenesis, The State Key Laboratory of Respiratory Disease, Guangzhou Medical College, Guangzhou, People's Republic of China; University of Bergen, Norway

## Abstract

**Background:**

Nuclear factor κB (NFκB) plays a key role in the regulation of apoptosis. The function of NFκB is inhibited by binding to NFκB inhibitor (IκB), and disruption of the balance of NFκB and IκB is related to the development of many diseases, including tumors. Therefore, we hypothesized that the *NFκB1 (-94del/insATTG)* and *NFκBIA* (2758 A>G) polymorphisms were associated with colorectal cancer (CRC) susceptibility.

**Methods:**

In a hospital-based case–control study of 1001 CRC patients and 1005 cancer-free controls frequency matched by age and sex, we genotyped polymorphisms using polymerase chain reaction-restriction fragment length polymorphism (PCR-RFLP) method and performed luciferase assays and Western blotting analysis to identify whether genetic variants in *NFκBIA* alter its gene expressions and functions and thus cancer risk.

**Results:**

We found that both *NFκB1-94 ins/delATTG* and *NFκBIA 2758 A>G* polymorphisms were correlated with CRC risk (OR = 1.47; 95%CI = 1.14–1.86, and OR = 1.38; 95% CI = 1.14–1.66, respectively). Furthermore, when evaluated these two polymorphisms together, the combined genotypes with 2 variant (risk) alleles (*2758GG* and *-94ins/ins+del/ins*) were associated with an increased risk of CRC (OR = 1.71; 95% CI = 1.23–2.38) compared to 0 variant, and the significant trend for 2 variant (risk) alleles were more pronounced among subgroups of aged <60 years, women, never drinkers, never smokers, persons with a normal BMI and those with a family history of cancer(*P^trend^*<0.01). Moreover, luciferase assays showed that the G allele in the 3′UTR significantly decreased *NFκBIA* mRNA stability and the *A* allele regulation by *miRNA449a in vitro* and that the NFκBIA protein expression levels of the *AA+AG* variant carriers were significantly higher in peritumoral tissues than those of the *2758GG* genotype.

**Conclusion:**

*NFκB1* and *NFκBIA* polymorphisms appear to jointly contribute to risk of CRC. These two variants may be a genetic modifier for CRC susceptibility in this southern Chinese population.

## Introduction

Colorectal cancer (CRC) is the third most common cancer in men and the second most common cancer in women around the globe, and it is estimated that there were approximately 1.2 million newly diagnosed CRC cases and 608,700 related deaths in 2008 [1]. Records from the municipal death registry of the city of Guangzhou, Guangdong, China, indicate that CRC is the fifth most common cancer. The mortality rate was dramatically increased from 4.33/10^5^ persons in 1970's to 12.13/10^5^ persons in 2000's [2]. The majority of CRC cases (approximately 80%) are sporadic [3], but a hereditary predisposition is present in 20–35% of patients, suggesting that both genetic and environmental factors contribute to CRC development [4]. Alcohol drinking and tobacco use [5], [6], dietary and lifestyle factors [7], and inflammatory bowel disease such as ulcerative colitis [8], [9] have shown to be associated with CRC risk. Although many people are exposed to these risk factors, only some of the exposed individuals develop CRC, indicating that genetic variation partly determines individual susceptibility to colorectal tumorigenesis.

Apoptosis, a highly regulated cellular process, participates in development, tissue homeostasis maintenance and elimination of unwanted cells [10]. Dysregulation in this process is likely to contribute to tumorigenesis [11]. The biochemical pathways of apoptosis are complicated and depend on not only the cells but also the inducers of apoptosis. Substantial evidence suggests that the occurrence and development of cancer is associated with both extended cell survival and suspended apoptosis in precancerous lesions and, consequently, aberrant apoptosis may allow for unchecked cell growth [12].

Nuclear factor kappa B (NFκB) is a major transcription regulator of the immune response, cell adhesion, differentiation, proliferation, and apoptosis [13]. Five members(p50/p105, p65/RelA, c-Rel, RelB, and p52/p100) in the NFκB family have been identified, and the dimeric form of NFκB1 p50/RelA is the major form [14]. In the resting cell, NFκB is inactivated in the cytoplasm through association with a sequestering inhibitory protein, IκBα, β or γ, and the most common protein of this family is the NFκB inhibitor α (NFκBIA) [15]. In the classical activation pathway, the phosphorylation and degradation of the inhibitory proteins lead to NFκB dissociation from the NFκB complex and translocation to the nucleus, where it can activate the transcription of a large number of genes [16]. As an important transcription factor, NFκB mediates the survival response by inhibiting p53-dependent apoptosis and up-regulating anti-apoptotic members of the Bcl-2 family and caspase inhibitors [17], [18]. In contrast, NFκB is also activated by both the extrinsic and intrinsic apoptotic stimuli and mediates upregulation of pro-apoptotic genes such as *TRAIL R2/DR5*, *Fas*, and *Fas* ligand [19], [20]. An inappropriate activation of NFκB could disturb tissue homeostasis and lead to dysregulated apoptosis. Furthermore, activity of NFκB has been observed in several types of cancers including CRC [21], [22], indicating it may play an important role in tumorigenesis [23], [24].

*NFκB1* (encoding for NFκB) maps to chromosome 4q23–q24 and consists of 24 exons [25], [26], and its inhibitory gene *NFκBIA* (encoding for IκB) is 3.5 kb long, with six exons, and is located on chromosome 14q13 [27], [28]. Genetic studies have identified single nucleotide polymorphisms (SNP) in *NFκB1* and *NFκBIA* [29], [30]. Recently, a common insertion/deletion (-94 insertion/deletionATTG rs28362491) polymorphism in the *NFκB1* promoter region and a 3′ -untranslated region (3′UTR) polymorphism 2758A>G (rs696) in *NFκBIA* were observed to be significantly correlated with inflammatory bowel disease [31], [32] and cancers [33], [34], [35]. Epidemiological studies have also investigated the association between *NFκB1* polymorphisms and risk of CRC in Germans and *NFκBIA* polymorphism and risk of CRC in the Swedish with conflicting results [36], [37].

There has been no previous report on the association between *NFκB1* and *NFκBIA* polymorphisms and CRC risk. As the NFκB/IκB system plays an important regulatory role in the apoptotic pathway and dysregulated expression of the *NFκB1* and *NFκBIA* has been observed in CRC, we hypothesized that combined *NFκB1* and *NFκBIA* polymorphisms may be associated with increased risk of CRC. To test this hypothesis, we genotyped the *NFκB1-94 insertion/deletionATTG* and *NFκBIA 2758A>G* polymorphisms in our ongoing hospital-based case–control study of CRC in a southern Chinese population, and further performed luciferase assays and Western blotting analysis to identify whether genetic variants in *NFκBIA* alter its gene expressions and functions and thus cancer risk.

## Materials and Methods

### Ethics statement

The study protocol was approved by the institutional review boards of Sun Yat-Sen University. Written informed consent was obtained from each participant after a full explanation of the study.

### Study subjects and sample collection

From July 2002 to April 2010, a total of 1001 patients with histopathologically-confirmed and untreated sporadic CRC were prospectively recruited from the First and Sixth Affiliated Hospital (Gastrointestinal & Anal Hospital) and Cancer Center of Sun Yat-Sen University, Guangzhou, China, the Affiliated Tumor Hospital of Guangzhou Medical College, Guangzhou, China, Guangdong Provincial People's Hospital, Guangzhou, China, and Panyu People's Hospital, Guangzhou, China. All these subjects were genetically unrelated ethnic Han Chinese and were from the city of Guangzhou and surrounding regions in southern China. Of the 1001 cases included in this study, there were 169 (16.9%) cases of right colon cancer, 309(30.9%)cases of left colon cancer, and 523 (52.2%) rectal cancer. According to American Joint Committee on Cancer staging Manual, there were 171 (17.1%)cases of stage I, 320 (31.9%)stage II, 345 (34.5%)stage III, and 165 (16.5%)stage IV. In the interim, a total of 1005 cancer-free controls were randomly selected from a subject pool of more than 10,000 individuals who participated in health check-up programs at the community health stations in Guangzhou, China.

The study participants were interviewed and data on smoking status, alcohol use and other factors including family history of cancer were obtained using a structured questionnaire. Smoking status, alcohol use and family history of cancer were defined as described previously [38]. Subjects whose body mass index (BMI) was <18.5 kg/m^2^ were categorized as being underweight, subjects whose BMI was from 18.5 to 24.0 kg/m^2^ were normal body weight, those who have a BMI >24.0 kg/m^2^ were overweight [39]. Cases belonging to familial adenomatous polyposis and those cases that fulfilled the criteria of Amsterdam for hereditary nonpolyposis CRC were excluded.

### Genotyping

Five mL blood was collected from each participant and genomic DNA was extracted using the DNA Blood Mini Kit (Qiagen, Valencia, CA) according to the manufacturer's instructions. Genotyping was performed by the polymerase chain reaction-restriction fragment length polymorphism (PCR-RFLP) method. For determination of the *NFκB1* promoter (rs28362491) polymorphism, the SNP-containing fragment was amplified using the following primers: 5′-TGGGCACAAGTCGTTTATGA-3′ (forward) and 5′-CTGGAGCCGGTAGGGAAG-3′ (reverse). PCR was run at 94°C for 3 min followed by 30 cycles of 94°C for 30 s, 56°C for 30 s, and 72°C for 60 s with a final extension at 72°C for 10 min. The PCR products (281/285 bp in size) were digested with *PfI*MI (Fermentas, Vilnius, Lithuania) at 37°C overnight followed by 2% agarose gel electrophoresis. For determination of the *NFκBIA* (rs696) polymorphism, the SNP-containing fragment was amplified using the following primers: 5′-GGCTGAAAGAACATGGACTTG-3′ (forward) and 5′-GTACACCATTTACAGGAGGG -3′ (reverse). The PCR was run at 94°C for 5 min followed by 32 cycles of 94°C for 30 s, 54.3°C for 45 s and 72°C for 60 s with a final extension at 72°C for 10 min. The amplified fragments were digested with *Hae*III (Fermentas, Vilnius, Lithuania) overnight at 37°C followed by 2% agarose gel electrophoresis.

Genotype analysis was done by two experimenters independently who were blinded to the status of the subjects as patient or control. To further validate the genotyping results, we randomly selected 10% samples for each of the 2 SNPs to perform repeat assays, and the results were 100% concordant. Additionally, 5% of the PCR products for each target genotype were purified and confirmed by direct sequencing (Figure S1 and Figure S2).

### Bioinformatics analysis

To investigate whether genetic variants of *NFκBIA* could bind to microRNAs (miRNA), we searched for target microRNAs of genetic variants of *NFκBIA* using the algorithm programs (http://www.targetscan.org and http://microrna.sanger.ac.uk/cgi-bin/targets/v5/search.pl).

### Cell culture

Three human colorectal adenocarcinoma cell lines, HCT116, HT29 and SW480 were purchased from Culture Collection of Chinese Academy of Science (Shanghai, China) and routinely cultured in Dulbecco's Modified Eagle's Medium or RPMI 1640 medium supplemented with 100 units/ml of penicillin, 100 µg/ml of streptomycin, and 10% fetal bovine serum (FBS). The cells were grown at 37°C with 5% CO2 in a humidified incubator.

### RNA interference, transient transfections and luciferase assays

*MiR-449a* mimics targeting *NFκBIA*, 5′-UGGCAGUGUAUUGUUAGCUGGU-3′ (sense) and 5′-CAGCUAACAAUACACUGCCAUU-3′ (anti-sense), *miR-449a* inhibitor, 5′-ACCAGCUAACAAUACACUGCCA-3′, miR-34b mimics targeting *NFκBIA*, 5′-CAAUCACUAACUCCACUGCCAU-3′ (sense) and 5′-GGCAGUGGAGUUAGUGAUUGUU-3′ (anti-sense), and *miR-34b* inhibitor, 5′-AUGGCAGUGGAGUUAGUGAUUG-3′ were synthesized by GenePharma Co. (Shanghai, China). In addition, the 3′UTR of the *NFκBIA 2758A* allele (2575 to 2955 bp relative to the translation start site) containing the *miR-449a* and *miR-34b* binding site was amplified using the following two primers, 5′-CCGCTCGAGCAAAGGGGCTGAAAGAA-3′ (sense) and 5′-AAGGAAAAAAGCGGCCGCAAAATGTGGTCCTTCCATGA-3′ (anti-sense). The amplified fragments were restricted with *Xho*I and *Not*I (New England BioLabs, Ipswich, MA) and then ligated into the *Xho*I and *Not*I restriction site of the dual-luciferase plasmid, psiCHECK™-2 (Promega, Madison, WI). Additionally, the 3′-UTR of the *NFκBIA 2758G* allele, which contained a mutated mRNA binding site, was amplified using PCR site-directed mutagenesis and cloned into psiCHECK™-2. For transfection, cells were seeded onto 24-well plates at 1×10^5^ cells/well and, after an overnight incubation, the cells were co-transfected with the reporter plasmid (2758G or 2758A allele ) and miR mimics or inhibitors using Lipofectamine 2000 (Invitrogen, Carlsbad, CA) following the manufacturer's protocol. Cells were lysed 48 h after transfection, and luciferase activity was measured using a Dual-Luciferase Reporter Assay System (Promega, Madison, WI). Each experiment was done in triplicate and at least three times independently.

### Western blotting analysis

To analyze the correlation between *NFκBIA* polymorphisms in its 3′UTR (2758A>G) and NFκBIA protein expression levels in tissues, 32 paired tumor and peritumoral tissues were obtained from sporadic CRC patients resected at the Sixth Affiliated Hospital of Sun Yat-Sen University and archived in the tumor bank. All tissues samples were histologically confirmed. Immunoblotting assays were performed as previously described [38] and antibodies against NFκBIA and β-actin (Precision Task Group, Chicago, USA) were used for the procedure. Protein densitometry was performed using Gel-Pro Analyzer 4.0 software (Media Cybernetics, Inc., Silver spring, MD) and NFκBIA expression was normalized against β-actin.

### Statistical analysis

Two-sided chi-square tests were used to assess differences in the distributions of age, sex, smoking status, alcohol use, BMI, menstruation history, and family history of cancer between cases and controls. The Hardy-Weinberg equilibrium (HWE) was tested by a goodness-of-fit chi-square test to compare the expected genotype frequencies with observed genotype frequencies (p2+2pq+q2 = 1) in cancer-free controls. The association between case-control status and each SNP, measured by the odds ratio (OR) and its corresponding 95% confidence interval (CI), was estimated using an unconditional logistic regression model, with and without adjustment for age, sex, smoking status, alcohol drinking status, BMI, tumor site, and family history of cancer. Recent studies indicate that an analysis of the combined genotypes might be more scientifically significant than an analysis of a single polymorphism in predicting the disease associations. Therefore the combined genotype data were further stratified by age, sex, and smoking status, alcohol drinking status, BMI, tumor site, and family history of cancer. Logistic regression modeling was also used for the trend test. Student's t test was done to examine the difference in levels of luciferase reporter gene expression between different constructs. Kruskal-Wallis one-way ANOVA tests were used for analyzing NFκBIA protein expression in peritumoral tissues of different genotypes. All tests were two-sided by using the SAS software (version 9.1; SAS Institute, Cary, NC). *P*<0.05 was considered statistically significant.

## Results

### Characteristics of the study population

The distribution of demographic characteristics of the 1001 sporadic CRC cases and 1005 cancer-free controls are shown in **Table 1**. Overall, no statistically significant difference was observed in age, sex, and smoking status between the cases and controls (*P*>0.05 in all). Compared with controls, there were more ever drinkers (cases vs. controls, 45.5% vs. 23.6%) (*P*<0.01) in the cohort of CRC cases. Moreover, compared with controls, CRC cases were more likely to have a family history of cancer or a higher BMI (*P*<0.05 in both). Consequently, these variables were further adjusted for in the multivariate logistic regression model to avoid possible confounding on the main effects of the SNPs under study. They were also used in the subsequent stratification and gene-environment interaction analysis.

**Table 1:** Frequency distributions of selected variables in CRC patients and cancer-free controls.

Variables	1001 Patients (n, %)		1005 Controls (n, %)		*P* a
Age (years)					0.4831
<50	233	23.3	231	23.0	
50–60	277	27.7	302	30.0	
>60	491	49.0	472	47.0	
Sex					0.9487
Male	604	60.3	605	60.2	
Female	397	39.7	400	39.8	
Smoking status					0.1964
Ever	482	48.1	455	45.3	
Never	519	51.9	550	54.7	
Alcohol drinking					<.0001
Ever	456	45.5	237	23.6	
Never	545	54.5	768	76.4	
Family history of cancer					0.0122
Yes	115	11.5	82	8.2	
No	886	88.5	923	91.8	
BMI(kg/m^2^)					0.0290
<18.5	63	6.3	39	3.9	
18.5–24.0	662	66.1	702	69.8	
>24.0	276	27.6	264	26.3	
Tumor site					
Right colon	169	16.9			
Left colon	309	30.9			
Rectum	523	52.2			
Tumor Stages					
I	171	17.1			
II	320	31.9			
III	345	34.5			
IV	165	16.5			

a *P* values for a two-sided χ^2^ test.

### PCR-RFLP study of the *NFκB1* promoter region and the *NFκBIA* 3′UTR region

**Figure 1:**
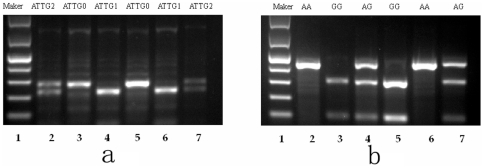
Image of the cleavage products of the *PfI*MI and *Hae*III restriction enzyme on 2% agarosegel. (**a**) *PfI*MI digestion band profile of the *NFκB1* gene on 2% agarosegel. Lane 1 Gene Ruler 100 bp Low Range Ladder, Lanes 2 and 7 del/ins heterozygote genotype, Lanes 4 and 6 *ins/ins* genotype, Lanes3, 5 *del/del* genotype. (**b**) *Hae*III digestion band profile of *NFκBIA* on 2% agarosegel. Lane 1 Gene Ruler 100 bp Low Range Ladder, Lanes 3, 5 homozygote G/G genotype, Lanes 2 and 6 homozygote A/A genotype, Lanes 4, 7 heterozygote A/G genotype.

We carried out analysis of the *NFκB1* promoter region by the PCR-RFLP method. Two ATTG repeats are present at the *NFκB1* promoter region, and one allele that has an ATTG insertion (ins) was cleaved into a 45-bp and a 240-bp fragment after digestion with *PfI*MI while the other deletion allele (del) that has only one ATTG at its promoter was not cleaved (Figure 1.a). Furthermore, after digestion with *Hae*III, the *2758GG* genotype produced a 316-bp and a 108-bp band whereas the 2758AA genotype produced a single 424-bp band, and heterozygotes displayed all 3 bands (Figure 1.b).

### The *NFκB1-94ins/delATTG* (rs28362491) and *NFκBIA 2758A>G* (rs696) polymorphisms are associated with the risk of sporadic CRC

The genotype and allele distributions of the *NFκB1-94ins/delATTG* (rs28362491) and *NFκBIA 2758A>G* (rs696) polymorphisms among the cases and controls are summarized in Table 2. The observed genotype frequencies of these two polymorphisms were all in agreement with the Hardy–Weinberg equilibrium in the control subjects (*P*>0.05). As shown in **Table 2**, for rs28362491, the distribution of the co-dominant genetic model (del/del vs. ins/ins vs. del/ins), and the dominant model (ins/ins+del/ins vs. del/del) differed significantly between CRC cases and controls (ins/ins: OR = 1.70; 95% CI = 1.29–2.25; *P*<0.01; del/ins: OR = 1.33; 95% CI = 1.03–1.74; *P*<0.01; ins/ins+del/ins: OR = 1.47; 95% CI = 1.14–1.86; *P*<0.01, respectively). For rs696, there was a significant difference in the distribution of the rs696 genotypes between CRC cases and controls (*P*<0.01). Significant differences were also noted in the distribution of the recessive model of rs696 (GG versus AA+AG) between CRC cases and controls (OR = 1.38; 95% CI = 1.14–1.66; *P*<0.05). Consistently, significant association was found between the two SNPs and the risk of sporadic CRC.

**Table 2:** Distribution of genotypes in *NFκB1* and *NFκBIA*, and results of logistic regression analysis for associations with risk of colorectal cancer.

Genotypes	Patients n (%)	Controls^a^ *n* (%)	*P* b	Crude OR (95% CI)	Adjusted OR (95% CI)^c^
Total no. of subjects	1001	1005			
Total no. of alleles	2002	2010			
*NFκB1* Rs28362491			0.0008		
del/del	138 (13.8)	186 (18.5)		1.00 (ref.)	1.00 (ref.)
del/ins	500 (49.9)	522 (51.9)		1.29 (1.01–1.66)	1.33 (1.03–1.74)
ins/ins	363 (36.3)	297 (29.6)		1.65 (1.26–2.15)	1.70 (1.29–2.25)
			0.004		
ins/ins+del/ins	863 (86.2)	819 (81.5)		1.42 (1.12–1.81)	1.47 (1.14–1.86)
del/del	138 (13.8)	186 (18.5)		1.00 (ref.)	1.00 (ref.)
*NFκBIA* Rs696 A>G			0.0075		
AA	233 (23.3)	212 (21.1)		1.00 (ref.)	1.00 (ref.)
GA	460 (45.9)	531 (52.8)		0.79 (0.63–0.99)	0.78 (0.62–0.98)
GG	308 (30.8)	262 (26.1)		1.07 (0.83–1.37)	1.04 (0.81–1.35)
			0.0196		
AA+GA	693 (69.2)	743 (73.9)		1.00 (ref.)	1.00 (ref.)
GG	308 (30.8)	262 (26.1)		1.38(1.15–1.65)	1.38(1.14–1.66)
Number of variant genotypes^d^			0.0008		
0	100 (10.0)	127 (12.6)		1.00 (ref.)	1.00 (ref.)
1	631 (63.0)	675 (67.2)		1.19 (0.89–1.58)	1.22 (0.90–1.63)
2	270 (27.0)	203 (20.2)		1.69 (1.23–2.32)	1.71 (1.23–2.38)
Trend test P value				0.0003	0.0004

a The observed genotype frequencies among the control subjects were in agreement with the Hardy-Weinberg equilibrium (*p* ^2^+2*pq*+*q* ^2^ = 1) (*χ^2^* = 3.539, P = 0.060 for Rs696G>A, P = 0.102 for Rs28362491 -94del/ins ATTG).

b Two-sided χ^2^-test for the distribution of genotype frequency.

c ORs were adjusted in a logistic regression model that included age, sex, smoking status, alcohol use, family history of cancer and BMI.

d Either the Rs696GG or Rs28362491 ins/ins+del/ins genotypes are risk genotypes as one.

### Stratification analysis of the association of combined *NFκB1* and *NFκBIA* polymorphisms with risk of CRC

We further analyzed the combined genotypes of the *NFκB1* and *NFκBIA* polymorphisms by examining age, sex, smoking, drinking, BMI, tumor site, and family history of cancer by logistic regression. We combined the *NFκB1* and *NFκBIA* polymorphisms based on the number of variant (risk) alleles (i.e., *2758GG* and *-94ins/ins+del/ins*). As shown in **Table 3**, compared with the *NFκB1-94 del/del* and *NFκB1IA 2758AA+AG* genotype, one variant combined genotype carriers who were <60 years of age had a higher risk of CRC (OR = 1.57; 95% CI = 1.04–2.38) (*P*<0.05). Further stratification analysis revealed that individuals with two variants had a significantly higher risk of CRC among subgroups of patients aged <60 years (OR = 2.18; 95% CI = 1.37–3.44) (*P*<0.01), women (OR = 2.36; 95% CI = 1.35–4.10) (*P*<0.01), never smokers (OR = 2.05; 95% CI = 1.30–3.22) (*P*<0.01), never drinkers (OR = 1.87; 95% CI = 1.23–2.84) (*P*<0.01), persons with a normal BMI (OR = 1.80; 95% CI = 1.21–2.69) (*P*<0.01) and those with a family history of cancer (OR = 4.57; 95% CI = 1.34–15.6, *P*<0.05).

**Table 3:** Stratification analysis of the variant number of genotypes by selected variables in colorectal cancer patients and controls.

	Patients (*n* = 1001)			Controls (*n* = 1005)			Adjusted OR ( 95% CI)^a^			
	Number of variant genotypes^c^			Number of variant genotypes^c^						
	0	1	2	0	1	2	0	1	2	*P*_trend_ b
	*n* (%)	*n* (%)	*n* (%)	*n* (%)	*n* (%)	*n* (%)				
Age (years)										
≤60	47 (9.2)	328(64.3)	135(26.5)	78 (14.6)	356(66.8)	99(18.6)	1.00 (ref.)	1.57(1.04–2.38)	2.18(1.37–3.44)	0.0015
>60	53 (10.8)	303(61.7)	135(27.5)	49 (10.4)	319(67.6)	104(22.0)	1.00 (ref.)	0.89(0.56–1.41)	1.28(0.77–2.11)	0.0923
Sex										
Male	67 (11.1)	376 (62.2)	161(26.7)	81(13.4)	393(65.0)	131(21.6)	1.00 (ref.)	1.36 (0.90–2.05)	1.49(0.98–2.27)	0.0530
Female	33 (8.3)	255(64.2)	109(27.5)	46(11.5)	282(70.5)	72(18.0)	1.00 (ref.)	1.30(0.80–2.11)	2.36(1.35–4.10)	0.0007
Smoking status										
Never	46(8.9)	329(63.4)	144(27.7)	71(12.9)	370(67.3)	109(19.8)	1.00 (ref.)	1.34(0.89–2.00)	2.05(1.30–3.22)	0.0006
Ever	51(11.2)	302(62.7)	126(26.1)	56(12.3)	305(67.0)	9420.7)	1.00 (ref.)	1.09(0.68–1.73)	1.42(0.87–2.33)	0.0986
Drinking status										
Never	53(9.7)	338(62.0)	154(28.3)	94(12.2)	950(69.0)	144(18.8)	1.00 (ref.)	1.09(0.75–1.59)	1.87(1.23–2.84)	0.0002
Ever	47(10.3)	293(64.3)	116(25.4)	33(13.9)	145(61.2)	59(24.9)	1.00 (ref.)	1.41(0.85–2.34)	1.58(0.89–2.80)	0.3055
Family history of cancer										
YES	10(8.7)	72(62.6)	33(28.7)	15(18.3)	55(67.1)	12(14.6)	1.00 (ref.)	2.50(0.91–6.87)	4.57(1.34–15.6)	0.0110
NO	90(10.2)	559(63.1)	237(26.7)	112(12.1)	620(67.2)	191(20.7)	1.00 (ref.)	1.14(0.59–2.06)	1.54(1.09–2.17)	0.0048
Tumor site										
Right colon	15(8.9)	106(62.7)	48(28.4)	127(12.6)	675(67.2)	203(20.2)	1.00 (ref.)	1.18(0.82–1.69)	1.59(1.07–2.37)	0.0107
Left colon	32(10.4)	193(62.4)	84(27.2)	127(12.6)	675(67.2)	203(20.2)	1.00 (ref.)	1.18(0.77–1.81)	1.62(1.01–2.61)	0.0238
Rectum	53(10.1)	332(63.5)	138(26.4)	127(12.6)	675(67.2)	203(20.2)	1.00 (ref.)	1.42(0.79–2.55)	1.94(1.02–3.67)	0.0293
BMI(kg/m^2^)										
<18.5	11(17.5)	38(60.3)	14(22.2)	3(7.69)	28(71.8)	8(20.5)	1.00 (ref.)	0.28(0.06–1.36)	0.22(0.02–2.40)	0.5447
18.5–24.0	65(9.8)	414(62.5)	183(27.7)	91(13.0)	470(66.9)	141(20.1)	1.00 (ref.)	1.32(0.92–1.90)	1.80(1.21–2.69)	0.0022
>24.0	24(8.7)	179(64.9)	73(26.4)	33(12.5)	177(67.1)	54(20.4)	1.00 (ref.)	1.10(0.83–1.57)	1.84(0.95–3.54)	0.0644

a ORs were adjusted for age, sex, smoking status, and alcohol use, BMI and family history of cancer in a logistic regression models.

b Adjusted in a logistic regression model that included age, sex, smoking status, alcohol use, BMI and family history of cancer.

c The number represents the numbers of variants within the combined genotypes, ie.0 = no variant (risk) allele and 1–2 = 1–2variant (risk) alleles; the variant (risk) alleles used for the calculation were the -94 ins/ins+del/ins and 2758GG alleles.

### *MiR-449a* mimics suppressed the activities of the *NFκBIA* 2758 A>G polymorphism

**Figure 2:**
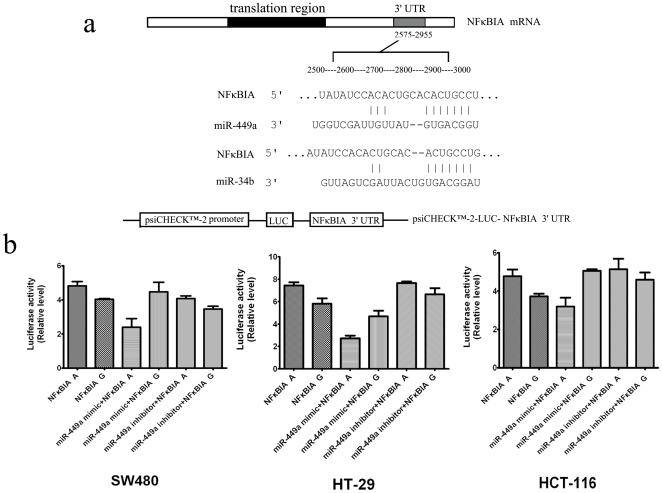
Validation of target microRNA predictions. (**a**) A putative target site of *miR-449a and miR-34b* highly conserved in the *NFκBIA* mRNA 3′UTR. (**b**) psiCHECK-2 Dual Luciferase Assay in three cell lines: SW480, HT29 and HCT116. Cells were transfected with reporter plasmids alone or co-transfected with microRNA. Luciferase expression was measured 48 hrs after transfection. The luciferase activity of each construct was normalized against the internal control of Renilla luciferase. Columns, mean from three independent experiments; bars, SD. *, *P*<0.05 for all comparisons of each cell line between the activities of the reporter gene constructs.

We further analyzed the 3′UTR of the *NFκBIA* 2758 A>G polymorphism using a computer algorithm and found that the polymorphism could affect miRNA binding. *MiR-449a* and *miR-34b*, which are involved in a wide variety of biological functions, were found to have a binding site within the 3′-UTR of the *NFκBIA* 2758 A>G polymorphism (Figure 2a). To determine the allele-specific effect of the *NFκBIA* 2758 A>G variant on the activity of the 3′UTR and whether this polymorphism affected miRNA binding, we transfected CRC cell lines SW480, HT29, and HCT116 with reporter plasmids carrying the *2758G* or *2758A* allele and miR mimics or inhibitors (Figure 2a). We found that, in the absence of miR mimics or inhibitors, compared with the *2758A* allele, the *2758G* allele exhibited decreased luciferase activities ( *P*<0.05)(Figure 2b). *MiR-449a* mimics could reduce the relative luciferase activities of the *2758A* allele while *miR-449a* inhibitors significantly up-regulate the relative luciferase activities of the *2758A* allele in all the three cell lines (*P*<0.01 for HT29; *P*<0.05 for SW480 and HCT116) (Figure 2b). On the other hand, *miR-34b* mimics failed to exert any noticeable effects on the relative luciferase activities of the *2758G* allele in these cell lines (data not shown). Taken together, these results demonstrated that the *2758G* allele in the 3′UTR of the *NFκBIA* reduced normalized luciferase activity compared to the *2758A* allele, likely corresponding to reduced mRNA stability or translational efficacy and that *miR-449a* have the ability to bind and partially repress luciferase expression via the *NFκBIA* 3′UTR segment when carrying the *2758A* allele of *NFκBIA* 2758 A>G polymorphism.

### Association of the *NFκBIA3′UTR* polymorphisms with the NFκBIA protein expression

**Figure 3:**
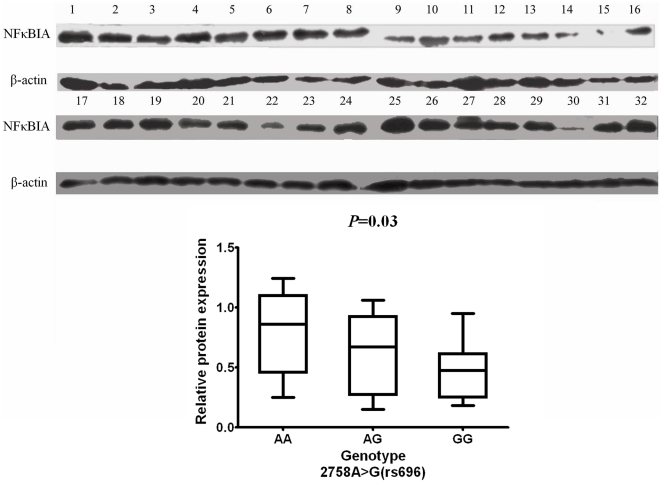
Association of the *NFκBIA* 3′UTR polymorphism with the NFκBIA protein expression. NFκBIA protein levels in 32 sporadic CRC peritumoral tissues from individuals with different genotypes of *2758A>G*. The NFκBIA protein expression levels were normalized to that of β-actin by calculating the relative expression levels. Individual genotype designation: For 2758A>G, lanes 1–3, 9–11, 17–20, 25–26, AA genotype (n = 12); lanes 4–6,12–14, 21–24, 27–28, GG genotype (n = 12); lanes 7–8, 15–16, 29–32, AG genotype (n = 8).

We were also interested in investigating whether the *NFκBIA3′UTR* polymorphisms (*2758A>G*) were associated with increased or reduced expression of NFκBIA protein. We collected 32 paired tumor and peritumoral tissues from the untreated CRC patients of different genotypes. Immunoblotting analysis revealed that the levels of NFκBIA (NFκBIA/β-actin protein ratio) in the peritumoral tissues of 12 cases of the *2758AA* genotype was 0.80±0.09 and those of 8 cases of the *2758AG* genotype was 0.63±0.11, both of which were significantly higher than those of the other 12 cases of the *2758GG* genotype (0.47±0.06) (analysis of variance test, *P*<0.05) (Figure 3). However, in the tumor tissues, NFκBIA expression levels did not differ significantly among the cases of different genotypes (data not shown). These results indicated that NFκBIA levels were significantly higher in peritumoral tissues from patients of the *2758AA+AG* genotypes than those of the *2758GG* genotypes.

## Discussion

In the present study, we investigated the associations of the *NFκB1-94del/insATTG* and *NFκB1IA 2758A>G* polymorphisms with risk of CRC in a southern Han Chinese population. We found that both the *NFkB1-94del/ins ATTG* and *NFkBIA 2758A>G* polymorphisms were associated with increased risk of CRC. For the *NFkB1*-*94del/insATTG* polymorphism, with the *-94del/del* as the reference, we found that the *-94(ins/ins+del/ins)* genotype was associated with a statistically significantly increased risk of CRC. For the *NFKBIA 2758A>G* polymorphism, with the *2758(AA+GA)* as the reference, we also found that the *2758GG* genotype was associated with a statistically significantly increased risk of CRC. Furthermore, when we evaluated *NFkB1* and *NFKBIA* polymorphisms in combination, we found that the combined *2758GG* and *-94ins/ins+del/ins* genotype was associated with a significantly increased risk of CRC compared with those without the *2758GG* and *-94ins/ins+del/ins* genotype, and this increased risk was more pronounced among younger than 60 years, women, never drinkers, never smokers, persons with a normal BMI and those with family history of cancer. In the in vitro assays, we also found that, compared with the *2758A* allele, the *2758G* variant allele showed significantly decreased mRNA stability and/or translational efficacy. Furthermore, we found that NFkBIA levels significantly higher in peritumoral tissues from patients of the *2758AA+AG* genotypes than those of the *2758GG* genotypes, suggesting that the *2758A>G* polymorphism is potentially functional and the polymorphism *2758A>G* at 3′UTR of *NFkBIA* could affect gene expression. To the best of our knowledge, this is the first study to investigate whether *NFkB1* and *NFkBIA* polymorphism and their combined polymorphism were associated with risk of CRC.

There are several lines of evidence supporting our findings. The *NFκB*1 pathways seem to play a critical role in multiple human pathologies by regulating the transcription of genes involved in the immune response, cell proliferation, and apoptosis [13]. It has been shown that alterations of *NFκB*1 expression plays an important role in the protection of cells from apoptosis [40]. *NFκB*1 activity has been observed in various types of cancer [41], including breast cancer [42] and CRC [21], to contribute to tumor angiogenesis and progression [43]. There are also many experimental data suggests that NFκB1/IκB pathway may participate in tumor cell invasion as well [44]. Therefore, the variants of the *NFκB1* and *NFκBIA* genes, if functional, could be expected to have an effect on cell death, and thus, carcinogenesis.

Several association studies have reported that the *NFκB1* and *NFκBIA* polymorphisms is related to the development of inflammatory and other diseases including ulcerative colitis, Graves' disease, and diabetes mellitus, and susceptibility to tumors including melanoma, bladder cancer and CRC in different ethnic groups [32], [36], [45], [46], [47], [48]. Gao *et al.*'s study reported a lack of an association between the *NFκBIA2758 A>G* polymorphism in northern Chinese population, but showed an association with the Swedish population and CRC risk [36]. Previous studies have provided evidence that the *del* allele may result in relatively decreased *NFκB*1 transcript levels and hence decreased p50/p105 *NFκB*1 protein production [32]. In our study, we further found that the change of the *2758 A* to *G* allele in the 3′UTR of *NFκBIA* decreased luciferase activities as assessed by luciferase assays. Our functional *in vitro* experiments suggested that *NFκBIA* 2758 *A>G* variants may affect mRNA stability. However, that the *NFκBIA* 2758 *A>G* variants affects translational efficacy or conduces to differential nuclear RNA processing or export also cannot be completely excluded [49]. Furthermore, it is well known that miRNAs can also cause mRNA cleavage or translational repression by forming imperfect base pairing with the 3′-UTR of target genes. In silico analysis of the NFκBIA mRNA sequence predicted the 2758 A>G variant of *NFκBIA* generates a potential seed site for *miR-449a* and our ex vivo luciferase data indicated that *miR-449a* reduced the relative luciferase activities via the NFκBIA 3′UTR target site created by the *2758A* allele. The results indicated that the *2758A* allele strengthens the binding of *miR-449a* with 3′UTR of *NFκBIA*, which in turn inhibits the expression of *NFκBIA*. Recent evidence indicate that miRNAs can bind to the 3′ UTRs of mRNAs and affect their translation, thus regulating cell proliferation, apoptosis and tumorigenesis [50]. Experimental studies concluded that SNPs located in miRNA-binding sites affect miRNA target expression and function, which is potentially associated with cancers [51]. This reconcile with our results that the *NFκBIA 2758A*-allele may enhance binding of *miR-449a* and affect *NFκBIA* gene expression and change of the *2758 A* to *G* allele in the 3′UTR of *NFκBIA* may affect mRNA stability or translational efficacy subsequent diminished protein levels. Consistently, we observed that NFκBIA protein expression was higher in the peritumoral tissues but not in tumor tissues from patients of the *2758AA+GG* genotype than those of the *2758GG* genotype. These data suggest that wildtype *A* allele may result in overexpression of *NFκBIA* in peritumoral normal tissues and hence increased NFκBIA protein production, resulting in inhibition of *NFκB* activity. However, carrying a mutant *2758G* allele result in relatively decreased *NFκBIA* mRNA stability and hence diminished NFκBIA protein production. As a major inhibitor of *NFκB*, decreased expression and dysfunction of *NFκBIA* may be a direct result of the activation of *NFκB* and thus cancer [16]. This is also consistent with our initial expectations, since CRC has been associated with increased levels of *NFκBIA G* allele. These findings indicate that the *NFκB1 -94del/ins ATTG* and *NFκBIA 2758 A>G* polymorphisms are functional. Our data from this relatively large sample size study further support the notion that the *NFκB1* and *NFκBIA* polymorphisms are potentially implicated in cancer risk.

In the present study, we also observed that the combined effect of the *NFκB1* and *NFκBIA* polymorphisms on risk of CRC was more pronounced among younger than 60 years, women, never drinkers, never smokers, persons with a normal BMI and those with a family history of cancer, suggesting that, in these subpopulations, gene-environment interaction may be very weak and the combined effect was an independent risk factor for these subpopulations. Compared with the published data, our results indicate that the genotype distributions of the *NFκB1* and *NFκBIA* polymorphisms vary with ethnicity. For example, the frequencies of the *del/del*, *del/ins*, and *ins/ins* genotypes of the *NFκB1-94del/insATTG* among our 1005 southern Han Chinese control subjects were 18.5%, 51.9%, and 29.6%, respectively, compared with 15.6%, 45.9%, and 38.4%, respectively, of 307 Germans in the study by Kathrin *et al.* [37]. Similarly, the frequencies of the *AA*, *AG* and *GG* genotypes of the *NFκBIA 2758A>G* in our controls were 21.2%, 52.8%, and 26.1%, respectively, compared with 6%, 45%, and 49%, respectively, of 109 cancer-free Australian controls in the study by Curran *et al.* [52]. However, the frequencies of the *del/del*, *del/ins*, and *ins/ins* genotypes of the *NFκB1-94del/insATTG* (18.5%, 51.9%, and 29.6%, respectively) were very similar to the published data by Sun *et al.* (17%, 58%, and 24%, respectively) [53]. In our southern Han Chinese controls, the frequency of the *NFκBIA* genotype distributions is also similar to that reported for that of the northern Han Chinese [36]. In this study, we also found that the *insertion* of *NFκB1-94del/insATTG* polymorphism increased CRC risk, which was contrary to the results reported by Andersen *et al* [54]. It is likely that the discrepancy results from the genetic difference in ethnicity (the frequencies of the del/del, del/ins, and ins/ins genotypes of the NFκB1-94del/insATTG among our 1005 southern Han Chinese control subjects were 18.5%, 51.9%, and 29.6%, respectively, compared with 13.5%, 45.9%, and 40.6%, respectively, of 756 Danes in the study). In addition, environmental effects such as dietary and lifestyle may also contribute to the discrepancy [7]. However, this hypothesis warrants further investigation.

In conclusion, our results suggested that both *NFκB1* and *NFκBIA* polymorphisms have effect on risk of CRC. These findings suggest that the *NFκB1* and *NFκBIA* polymorphisms may jointly contribute to the risk of CRC in a southern Chinese population, which were consistent with the functional assays we performed. Our study indicated that the *NFκB1-94(ins/ins+del/ins)* and *NFκBIA GG* polymorphism may be a genetic marker for susceptibility to CRC in Chinese populations. However, additional studies with more detailed data on environmental exposure and survival data are required to verify these findings. Therefore, future population-based studies are needed to verify the findings.

## Supporting Information

Figure S1 ***NFκB1-94ins/delATTG*** **genotyping by direct sequencing: (a)** ***del/del*** **genotype; (b)** ***del/ins*** **genotype; (c)** ***ins/ins*** **genotype.**
(PPT)

Figure S2 ***NFκBIA 2758A>G*** **genotyping by direct sequencing: (a)** ***GG*** **genotype; (b)** ***AA*** **genotype; (c)** ***AG*** **genotype.**
(PPT)
