# Supplementary material for: NFκB1 and NFκBIA Polymorphisms Are Associated with Increased Risk for Sporadic Colorectal Cancer in a Southern Chinese Population
Source: PLoS One. 2011 Jun 30;6(6):e21726. doi: 10.1371/journal.pone.0021726 (PMC3128094; doi:10.1371/journal.pone.0021726)
Supplement: Figure S2 — NFκBIA 2758A>G genotyping by direct sequencing: (a) GG genotype; (b) AA genotype; (c) AG genotype. (PPT) [file pone.0021726.s002.ppt]

## Slide 1
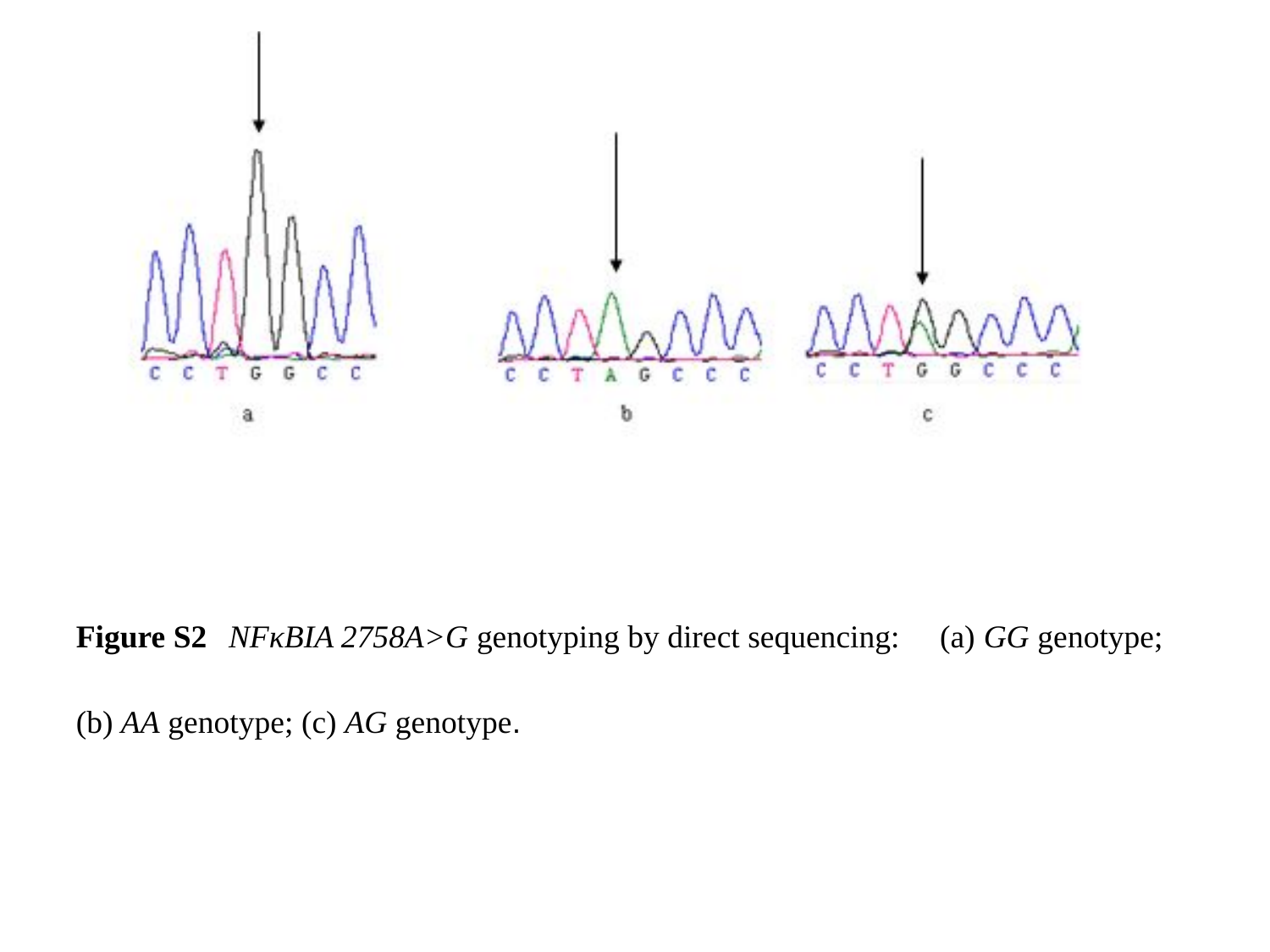

Figure S2 NFκBIA 2758A>G genotyping by direct sequencing: (a) GG genotype; (b) AA genotype; (c) AG genotype.
